# Bioelectrical impedance vector analysis in critically ill patients: a prospective, clinician-blinded investigation

**DOI:** 10.1186/s13054-015-1009-3

**Published:** 2015-08-12

**Authors:** Sarah L. Jones, Aiko Tanaka, Glenn M. Eastwood, Helen Young, Leah Peck, Rinaldo Bellomo, Johan Mårtensson

**Affiliations:** Department of Intensive Care, Austin Hospital, Austin Health, Heidelberg, Melbourne, VIC 3084 Australia; Australian and New Zealand Intensive Care Research Centre, School of Preventive Medicine and Public Health, Monash University, Melbourne, VIC Australia; Section of Anaesthesia and Intensive Care Medicine, Department of Physiology and Pharmacology, Karolinska Institutet, Stockholm, Sweden

## Abstract

**Introduction:**

Assessment of fluid status in critically ill patients is challenging. We aimed to assess the feasibility and validity of bioelectrical impedance vector analysis (BIVA) as a measure of hydration in critically ill patients.

**Methods:**

We performed twice-daily BIVA measurements and fluid balance calculations and recorded physiological variables in mechanically ventilated patients within 24 h of intensive care unit (ICU) admission for up to 5 days. Treating clinicians were blinded to BIVA results.

**Results:**

We performed 344 BIVA measurements in 61 patients. According to BIVA, 14 patients (23 %) were dehydrated, 22 (36 %) were normally hydrated and 25 (41 %) were overhydrated upon ICU admission. Patients with normal BIVA hydration were less sick, had fewer comorbidities and had less deranged physiology than patients found to be dehydrated or overhydrated with BIVA. Cumulative fluid balance increased in patients found to be dehydrated with BIVA by a mean of 3.4±2.2 L, whereas in patients found to be overhydrated with BIVA, it decreased by a mean of 4.5±6.9 L. In patients found to be normally hydrated with BIVA, fluid balance remained unchanged. BIVA-defined hydration increased with 1 L (median change 1.5 %, *P* =0.09) or 2 L (median change 0.7 %, *P* =0.09) of calculated fluid gains. BIVA-defined hydration decreased (median change −0.8 %, *P* =0.02) with a negative cumulative fluid balance of >2 L. BIVA-defined hydration between first and last measurement correlated with the corresponding change in fluid balance (ρ =0.25, *P* =0.05).

**Conclusions:**

BIVA is feasible in critically ill patients. Its validity is supported by the observed characteristics of patients with different degrees of BIVA hydration upon admission and by different fluid management of such patients by blinded clinicians. The sensitivity of repeated BIVA hydration measurements to detect fluid accumulation or fluid balance changes <2 L was low, however. These contradictory findings provide the rational basis for studies of BIVA-assisted fluid management in ICU patients.

**Electronic supplementary material:**

The online version of this article (doi:10.1186/s13054-015-1009-3) contains supplementary material, which is available to authorized users.

## Introduction

Fluid management in patients who are critically ill is challenging because their hydration status is difficult to assess at the bedside. Widely used markers such as invasively obtained intravascular pressures are acknowledged to have major flaws as measures of hydration [1–3], and they provide no information about extravascular or intracellular fluid status. Furthermore, calculated cumulative fluid balance during intensive care unit (ICU) admission may be inaccurate, does not account for insensible or third-space fluid losses and does not incorporate preadmission fluid status. Standardised body weight measurement, even using beds with electronic weight estimation, have repeatedly been shown to be divorced from cumulative fluid balance calculations [4–6]. Clinical detection of fluid overload as oedema is insensitive and requires the accumulation of 4–5 L before detection [7]. Finally, deuterium dilution studies, which are the gold standard for total body water (TBW) assessment, are not feasible on a daily basis in the ICU setting [8].

Bioelectrical impedance vector analysis (BIVA) is a rapid, non-invasive bedside technique to measure fat-free TBW that correlates closely with the deuterium dilution technique [9, 10]. BIVA has been reported to be useful to monitor hydration status during fluid removal in patients with decompensated heart failure [11, 12] and during intermittent haemodialysis [13–17]. Thus, it may also prove useful in patients who are critically ill.

However, there is a paucity of studies of the feasibility and validity of BIVA-derived measurements in critically ill patients, and no study to date has examined the association between BIVA-measured hydration status and changes in fluid balance or clinician-driven fluid management. Accordingly, we conducted a prospective, clinician-blinded, observational study to assess the feasibility and validity of BIVA in critically ill patients.

## Materials and methods

### Study design

We conducted a prospective, clinician-blinded, observational study from August 2014 to February 2015 to evaluate BIVA-measured hydration status in adult ICU patients at a tertiary hospital. The study was approved by the Austin Hospital Human Research Ethics Committee (reference number H2012/04864). Informed consent was obtained from the person legally responsible for the patient.

### Inclusion and exclusion criteria

We enrolled a consecutive convenience sample of screened patients by eligibility on weekdays only. We included adult patients (≥18 years) within 24 h of admission to the ICU who were mechanically ventilated and were expected to stay in the ICU for ≥48 h.

We excluded patients with end-stage kidney disease undergoing long-term dialysis, pregnant patients, patients admitted to the ICU following elective cardiac surgery, patients with pacemakers or implantable defibrillators and patients with diaphragmatic pacing.

### Study protocol

We performed twice-daily (morning and afternoon) BIVA measurements using Renal EFG BIVA™ Technology (EFG Diagnostic, Belfast, UK) during the first 5 days in the ICU or until ICU discharge. At the time of each BIVA measurement, we recorded mean arterial pressure (MAP), central venous pressure (CVP), vasopressor requirements, gas exchange, arterial lactate, creatinine and cumulative fluid balance.

Three trained operators obtained the BIVA measurements according to the manufacturer’s instructions with patients positioned horizontally and supine whenever possible for ≥2 minutes. BIVA results were not made available to treating clinicians at any time during the study.

### Principles of Renal EFG with BIVA™ Technology

BIVA provides a quantitative estimate of TBW as a percentage of fat-free body mass. It combines bioelectrical impedance with capacitance measures (i.e., time required to charge a circuit). Whole-body impedance is considered as a combination of resistance (R) and reactance (Xc). The arc tangent of Xc/R is called the *phase angle* and represents the phase difference between voltage and current [18].

BIVA is safe and non-invasive and can be performed at the bedside, giving a result within minutes. It is based on the electrical principle that the body is a circuit with a given resistance (opposition of current flow through intracellular and extracellular solution) and reactance (capacitance of the cells to store energy) [19]. Through the application of a 50-kHz current to the body via a pair of electrodes (one placed on the dorsum of the wrist and the other on the ipsilateral ankle), it gives a measurement of TBW in fat-free tissues.

The accuracy of the test rests on careful placement of the BIVA electrodes and the connecting cable clips. Standardised by height (H), BIVA results are displayed graphically, comparing R/H to Xc/H and reflecting hydration abnormalities and soft tissue mass. If obtained as a single measurement, the BIVA result can be compared with the normal population, represented on the graph by confidence ellipses, with data expected to fall within the reference 75 % tolerance ellipse. Relative hydration can also be depicted as vector length. Longer vectors equate to volume depletion, whilst shorter vectors correspond to volume overload [19].

**Fig. 1:**
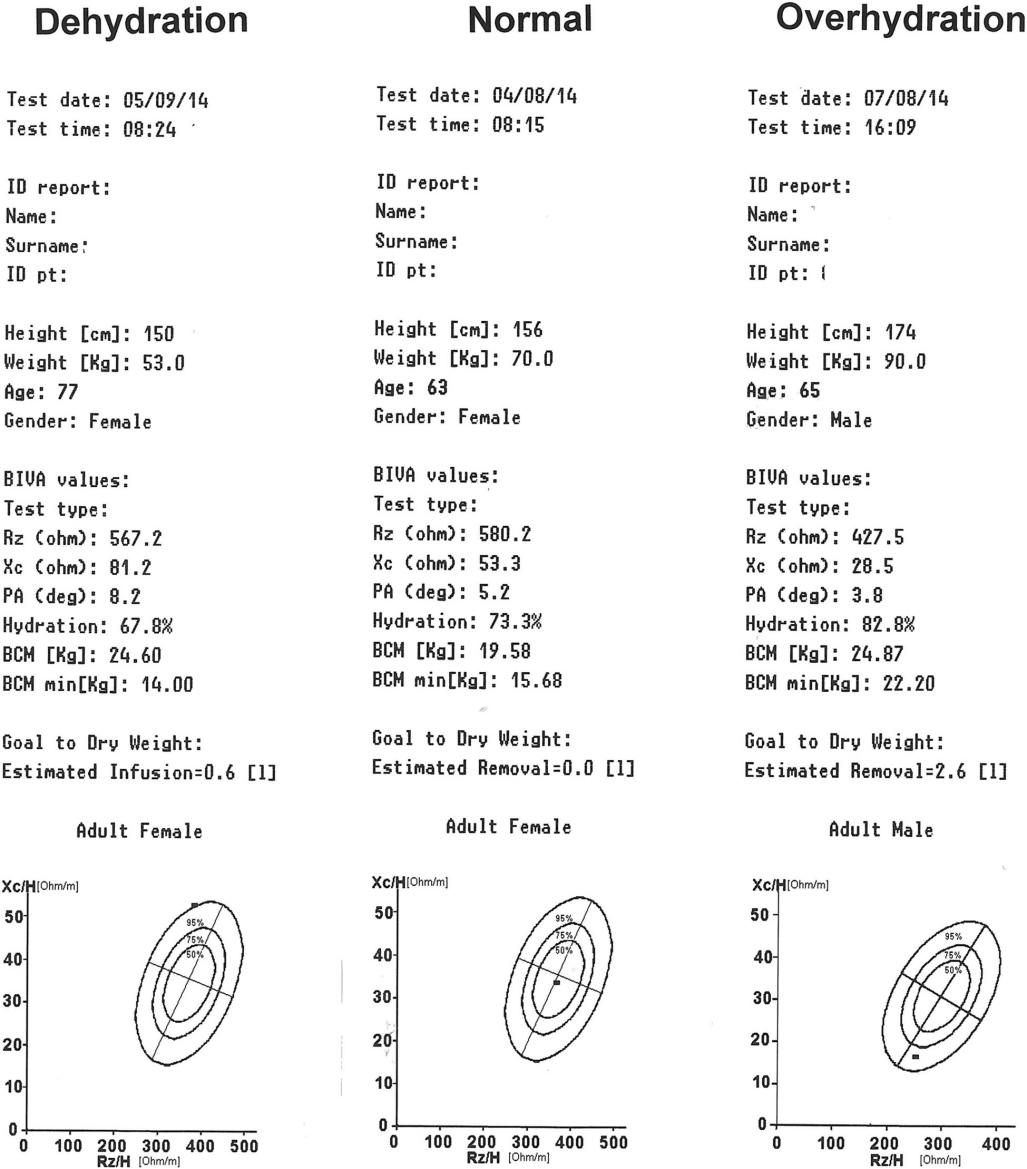
Examples of values obtained with the Renal EFG BIVA™ Technology. *BIVA* bioelectrical impedance vector analysis, *H* height, *PA* phase angle, *Xc* reactance, BCM body cell mass

In our study, patients were classified by BIVA as dehydrated (<72.7 % of fat-free body mass), normally hydrated (hydration 72.7–74.3 %) or overhydrated (hydration >74.3 %). Examples of values obtained with the Renal EFG BIVA™ Technology are displayed in Fig. 1.

### Statistical analysis

Statistical analyses were performed using STATA version 11.2 software (StataCorp, College Station, TX, USA). Continuous variables were summarised using median and interquartile range (IQR) or mean ± standard deviation, and categorical variables were expressed as number (%). The Kruskal-Wallis test was used for comparison between continuous variables, and the χ^2^ test or Fisher’s exact test was used for comparisons between categorical variables. Changes over time for multiple measures of cumulative fluid balance were tested by repeated-measures analysis of variance (RM-ANOVA) using ICU day as the RM variable. For comparison of cumulative fluid balance change over time between categories, an interaction variable (between category and time) was introduced in the RM-ANOVA model. The nonparametric Wilcoxon signed-rank test was used to assess the change between two prespecified time points. Spearman’s rank correlation coefficient was used to assess the relationship between changes in BIVA hydration, cumulative fluid balance, CVP and lactate. Intra-BIVA coefficient of variation between duplicate measurements obtained under steady-state conditions was calculated as described in Additional file 1. A *P* value <0.05 was considered statistically significant.

## Results

### Patient characteristics

**Fig. 2:**
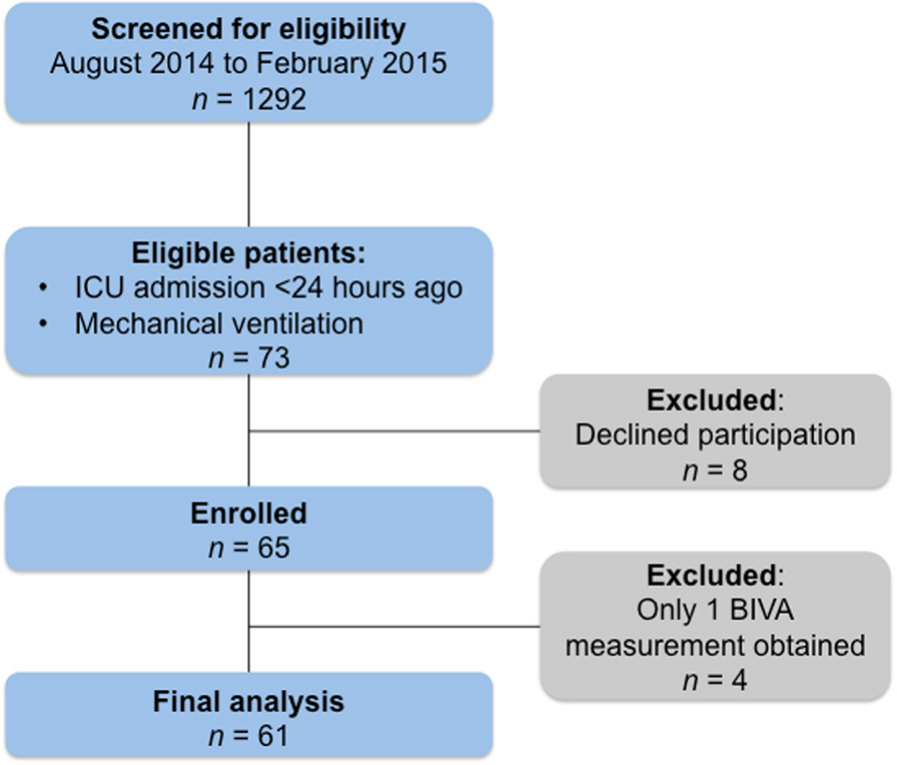
Selection of study patients. *BIVA* bioelectrical impedance vector analysis, *ICU* intensive care unit

We screened 1292 patients and identified 73 mechanically ventilated patients admitted to the ICU for <24 h (Fig. 2). The restrictive inclusion criteria explain the relatively low screening/inclusion ratio. Firstly, we did not include 231 patients admitted after cardiac surgery between August 2014 and February 2015. Secondly, approximately 50 % of patients admitted to the unit did not require mechanical ventilation and were therefore not eligible. Thirdly, we did not include patients admitted on weekends or during holidays. Finally, some patients were subject to multiple invasive procedures and investigations and therefore could not be included within 24 h of ICU admission. After excluding 8 patients who declined participation and 4 patients in whom only one BIVA measurement could be performed owing to death or early discharge from the ICU, 61 patients were included in the final analysis. We included these patients within a median of 14 (IQR 7 to 19) h and obtained a total of 344 BIVA measurements. Their median age was 63 years and the median Acute Physiology and Chronic Health Evaluation III score was 64 (Table 1).

**Table 1:** Baseline characteristics of the study cohort, stratified by hydration level according to bioelectrical impedance vector analysis measurement upon admission

	All	Dehydration	Normal	Overhydration	*P* value^a^
	(*n* =61)	(*n* =14)	(*n* =22)	(*n* =25)	
Age, yr	63 (48–72)	66 (56–76)	61 (43–68)	65 (52–75)	0.43
Female sex, *n* (%)	23 (38 %)	5 (38 %)	11 (50 %)	7 (28 %)	0.30
Height, cm	168 (158–175)	168 (160–170)	169 (158–175)	173 (159–178)	0.77
Weight, kg	76 (67–96)	75 (64–85)	74 (67–88)	90 (72–109)	0.16
Body mass index, kg/m^2^	28 (24–33)	26 (24–29)	27 (23–30)	29 (25–36)	0.22
APACHE III score	62 (47–82)	75 (49–82)	53 (45–67)	72 (48–92)	0.21
Time from ICU admission until enrolled, h	14 (7–19)	11 (5–19)	11 (4–19)	15 (12–19)	0.23
Comorbidity					
Cardiovascular, *n* (%)	26 (43 %)	4 (29 %)	11 (50 %)	11 (44 %)	0.51
COPD/asthma, *n* (%)	8 (13 %)	5 (38 %)	0 (0)	3 (12 %)	0.007
Diabetes mellitus, *n* (%)	16 (26 %)	1 (7 %)	5 (23 %)	10 (40 %)	0.08
Chronic liver disease, *n* (%)	8 (13 %)	1 (7 %)	0 (0)	7 (28 %)	0.009
Neurological, *n* (%)	3 (5 %)	1 (7 %)	1 (5 %)	1 (4 %)	1.0
Chronic kidney disease, *n* (%)	4 (7 %)	1 (7 %)	0 (0)	3 (12 %)	0.27
Malignancy, *n* (%)	9 (15 %)	3 (21 %)	3 (14 %)	3 (12 %)	0.74
Non-operative admission diagnosis					
Sepsis, *n* (%)	10 (16 %)	4 (29 %)	4 (18 %)	2 (8 %)	0.24
Cardiac, *n* (%)	8 (13 %)	4 (29 %)	2 (9 %)	2 (8 %)	0.21
Neurological, *n* (%)	9 (15 %)	1 (7 %)	5 (23 %)	3 (12 %)	0.47
Respiratory, *n* (%)	9 (15 %)	3 (21 %)	6 (27 %)	0 (0)	0.01
Intoxication, *n* (%)	2 (3 %)	0 (0)	1 (5 %)	1 (4 %)	1.0
Liver, *n* (%)	2 (3 %)	0 (0)	0 (0)	2 (8 %)	0.34
Gastrointestinal, *n* (%)	5 (8 %)	0 (0)	0 (0)	5 (20 %)	0.02
Operative admission diagnosis					
Neurological, *n* (%)	3 (5 %)	1 (7 %)	0 (0)	2 (8 %)	0.44
Vascular, *n* (%)	2 (3 %)	0 (0)	1 (5 %)	1 (4 %)	1.0
Gastrointestinal, *n* (%)	11 (18 %)	1 (7 %)	3 (14 %)	7 (28 %)	0.27

*APACHE III* Acute Physiology and Chronic Health Evaluation III, *BIVA* bioelectrical impedance vector analysis, *COPD* chronic obstructive pulmonary disease

Continuous variables are presented as median (interquartile range)

^a^Statistical comparisons of the data were performed using the χ^2^ test or Fisher’s exact test for categorical variables and the Kruskal-Wallis test for continuous variables

At the time of inclusion, BIVA identified 14 patients (23 %) as dehydrated, 22 (36 %) as normally hydrated and 25 (41 %) as overhydrated. The characteristics of study patients according to their level of BIVA hydration at inclusion are summarised in Table 1. Asthma and chronic obstructive pulmonary disease were more common among dehydrated patients than in the other patients (*P* =0.007). In contrast, chronic liver disease was more frequently observed in overhydrated patients than among the other patients (*P* =0.009). Similar findings applied to patients admitted with non-operative respiratory and gastrointestinal diagnoses.

### Physiological state on admission

BIVA values, biochemistry, haemodynamics and ventilatory variables at inclusion are summarised in Table 2. Upon admission, overhydrated patients required more noradrenaline than dehydrated and normally hydrated patients (*P* =0.02). Moreover, kidney dysfunction was more pronounced in overhydrated patients than in the other patients (*P* =0.03). Three dehydrated patients and nine normally hydrated patients were enrolled in the study before a central venous catheter was inserted. Admission CVP was therefore missing in these patients.

**Table 2:** Admission bioelectrical impedance vector analysis data, haemodynamics, oxygen exchange and biochemical data

	Dehydration	Normal	Overhydration	*P* value^a^
	(*n* =14)	(*n* =22)	(*n* =25)	
BIVA data				
Hydration, %	71.0 (64.2–72.6)	73.5 (73.2–73.9)	85.2 (78.4–89.0)	<0.001
Resistance/height, Ω/m	321 (249–348)	314 (257–351)	224 (188–267)	0.002
Reactance/height, Ω/m	43.6 (40.7–58.9)	29.6 (26.4–33.9)	16.5 (11.5–20.4)	<0.001
Phase angle, degrees	9.1 (7.3–10.3)	5.5 (5.2–6.3)	3.8 (3.0–4.4)	<0.001
Haemodynamic data				
Mean arterial pressure, mmHg	71 (67–76)	78 (70–92)	71 (67–78)	0.13
Central venous pressure, mmHg	11 (8–12)	10 (8–12)	12 (10–14)	0.08
Missing observations, *n*	3	9	0	
Noradrenaline, μg/min	3.5 (0–6)	0 (0–2)	6 (1–10)	0.04
Oxygen exchange				
FiO_2_, %	0.40 (0.25–0.50)	0.30 (0.21–0.40)	0.30 (0.21–0.40)	0.28
PaO_2_, mmHg	82 (67–98)	105 (75–122)	82 (72–97)	0.07
PaO_2_/FiO_2_ ratio, mmHg	249 (166–300)	334 (273–407)	316 (236–419)	0.06
Biochemical data				
Creatinine, μmol/L	72 (55–131)	70 (40–100)	116 (73–160)	0.03
Missing observations, *n*	1	0	0	
Sodium, mmol/L	138 (134–142)	137 (135–140)	137 (135–139)	0.97
Lactate, mmol/L	1.6 (1.1–2.2)	1.5 (1.1–2.4)	1.9 (1.4–2.5)	0.45

*BIVA* bioelectrical impedance vector analysis, *FiO*_*2*_ fraction of inspired oxygen, *PaO*_*2*_ partial pressure of arterial oxygen

Continuous variables are presented as median (interquartile range)

^a^Statistical comparisons of the data were performed using the Kruskal-Wallis test

### Changes in fluid balance and BIVA hydration

**Fig. 3:**
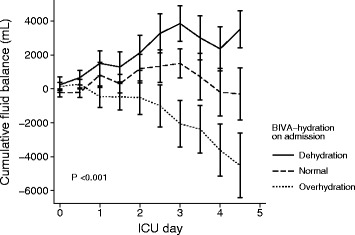
Cumulative fluid balance during the first 4 days in the intensive care unit (ICU) in patients with dehydration, normal hydration and overhydration on admission. Values are mean ± SE. *BIVA* bioelectrical impedance vector analysis

Changes in cumulative fluid balance according to BIVA hydration are displayed in Fig. 3. By day 4, blinded clinician driven cumulative fluid balance had increased in patients found to be dehydrated with BIVA (*P* =0.01 by RM-ANOVA) by a mean of 3.4±2.2 L. In contrast, the cumulative fluid balance in patients found to be overhydrated with BIVA had decreased by a mean of 4.5±6.9 L (*P* <0.001 by RM-ANOVA). In normally hydrated patients, the fluid balance remained unchanged. The components of the cumulative fluid balance (fluid input and output), urine output and loop-diuretic administration are displayed in the Additional file 1.

**Fig. 4:**
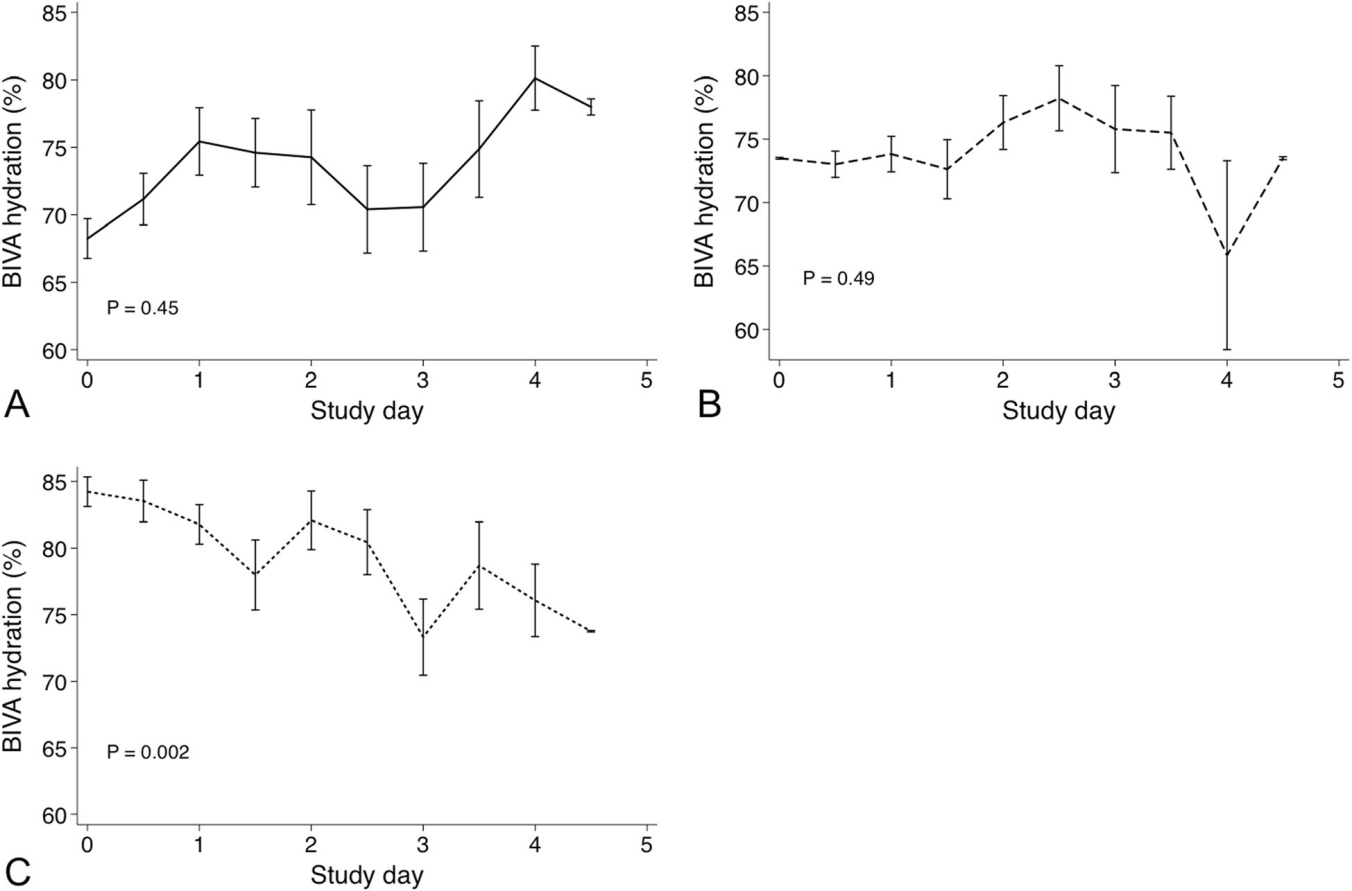
Mean daily hydration determined by bioelectrical impedance vector analysis (BIVA) in patients with dehydration (**a**), normal hydration (**b**) and overhydration (**c**) upon admission. *P* values represent repeated-measures analysis of variance for change in hydration level over time

A trend toward increased BIVA hydration over time was observed in patients classified as dehydrated upon admission (*P* =0.45). In contrast, BIVA hydration significantly decreased in patients with BIVA overhydration upon admission (Fig. 4).

### Relationship between changes in fluid balance and BIVA hydration

Of the 61 patients, 26 (43 %) reached a cumulative fluid balance of at least +1000 ml (median 1385 ml, IQR 1205–2022 ml) within a median of 1 day after the first BIVA measurement (Table 3). Simultaneously, median BIVA hydration also increased from 73.8 % to 79.7 % (*P* =0.09). During the same observation period, there was no significant change in CVP or lactate. In 13 (21 %) of 61 patients, cumulative fluid balance reached at least +2000 ml (median 2419 ml, IQR 2196–2696 ml) within the same time frame. The corresponding BIVA hydration also increased from 73.1 % to 73.8 % (*P* =0.09). However, no significant changes in CVP or lactate were observed. In the 19 patients (31 %) with a negative fluid balance of −1000 ml, a small decrease in BIVA hydration was seen (*P* =0.22). In patients with a negative fluid balance of at least −2000 ml, we observed a more pronounced decrease in BIVA hydration, from 77.3 % to 73.9 % (*P* =0.02). There were no significant corresponding directional changes in CVP or lactate.

**Table 3:** Changes in hydration determined by bioelectrical impedance vector analysis in patients with different magnitude changes in cumulative fluid balance

	First measurement	Second measurement^a^	Change between first and second measurement	*P* value^b^
≥1-L positive fluid balance, *n* =26				
Cumulative fluid balance, ml	0 (0)	1385 (1205–2022)	1385 (1205–2022)	
Study day	0 (0)	1.0 (1.0–2.0)	1.0 (1.0–2.0)	
Central venous pressure, mmHg	12 (10–13)	12 (8–15)	0 (−3 to 3)	0.84
Missing observations, *n*	7	7	7	
Lactate, mmol/L	2 (1.1–3.3)	1.7 (1.5–2.5)	−0.3 (−0.6, 0.4)	0.67
BIVA hydration, %	73.8 (72.6–82.1)	79.7 (72.9–86.0)	1.5 (−0.8 to 7.0)	0.09
≥2-L positive fluid balance, *n* =13				
Cumulative fluid balance, ml	0 (0)	2419 (2196–2696)	2419 (2196–2696)	
Study day	0 (0)	1.0 (1.0–2.0)	1.0 (1.0–2.0)	
Central venous pressure, mmHg	12 (10–14)	13 (11–17)	1.5 (0–3)	0.14
Missing observations, *n*	5	5	5	
Lactate, mmol/L	2.2 (1.2–3.7)	2 (1.7–2.4)	0.3 (0.2–0.7)	0.38
BIVA hydration, %	73.1 (72.6–73.9)	73.8 (73.3–80.3)	0.7 (0–6.4)	0.09
≥1-L negative fluid balance, *n* =19				
Cumulative fluid balance, ml	0 (0)	−1553 (−2056 to -1297)	−1553 (−2056 to −1297)	
Study day	0 (0)	1.5 (0.5–2.5)	1.5 (0.5–2.5)	
Central venous pressure, mmHg	11 (10–14)	12 (9–15)	0 (-2.5, 2)	0.72
Missing observations, *n*	3	3	3	
Lactate, mmol/L	1.5 (1.1–2.4)	1.6 (1.2–2.1)	0.1 (−0.4, 0.5)	0.88
BIVA hydration, %	77.3 (73.0–89.0)	76.5 (73.0–85.2)	−0.3 (−4.5 to 0.7)	0.22
≥2-L negative fluid balance, *n* =15				
Cumulative fluid balance, ml	0 (0)	−2426 (−2606 to −2228)	-2426 (−2606 to −2228)	
Study day	0 (0)	1.5 (1.0–2.5)	1.5 (1.0–2.5)	
Central venous pressure, mmHg	11 (10–13)	10 (8–12)	−2 (−5, 0)	0.11
Missing observations, *n*	2	2	2	
Lactate, mmol/L	2 (1.1–2.4)	1.5 (1.2–2.3)	−0.1 (−0.6, 0.3)	0.53
BIVA hydration, %	77.3 (73.3–88.3)	73.9 (72.7–85.2)	−0.8 (−3.4 to −0.1)	0.02

*BIVA* bioelectrical impedance vector analysis

Continuous variables are presented as median (interquartile range)

^a^Second measurement refers to the time when the pre-determined cumulative fluid balance was reached

^b^Change between the first and second measurement assessed by nonparametric Wilcoxon signed rank test

**Fig. 5:**
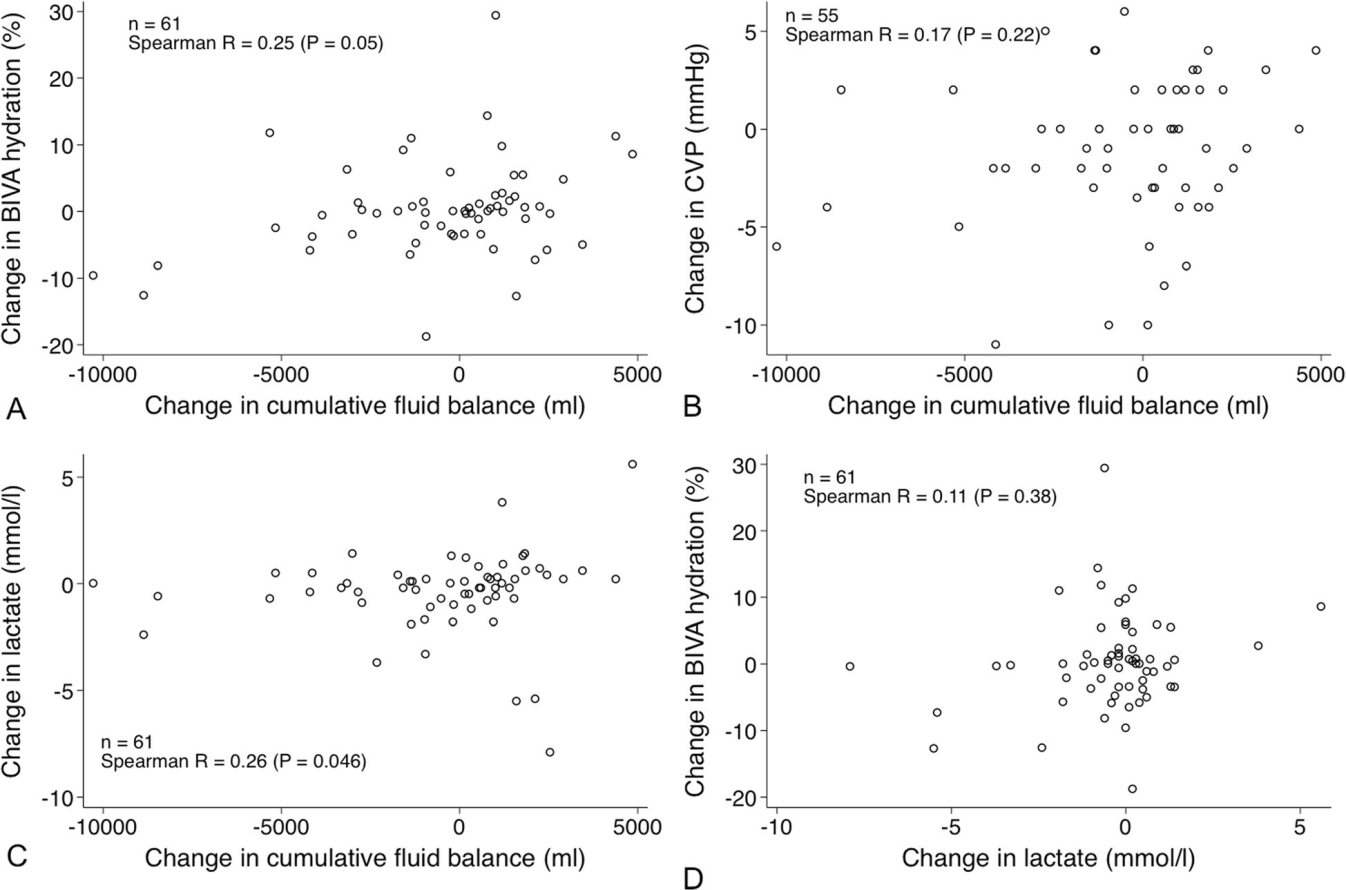
Changes in hydration determined by bioelectrical impedance vector analysis (BIVA) (**a**), central venous pressure (CVP) (**b**) and lactate (**c**) versus changes in cumulative fluid balance and changes in BIVA hydration versus changes in lactate (**d**) between the time points of the first and last BIVA recordings

We also considered the changes in BIVA hydration, CVP, lactate and cumulative fluid balance between the time points of the first and last BIVA recordings (Fig. 5). We found a significant but weak correlation between changes in fluid balance and changes in BIVA hydration, as well as changes in lactate, but no correlation with changes in CVP. In addition, we found no significant correlation between changes in BIVA hydration and changes in lactate.

Complete longitudinal data on BIVA hydration and cumulative fluid balance was available for two patients treated with continuous renal replacement therapy (CRRT; one of whom was treated simultaneously with extracorporeal membrane oxygenation). These patients’ BIVA and fluid balance trajectories are displayed in Additional file 1.

### Association of BIVA hydration and patient outcomes

Patient outcomes are shown in Table 4. A non-significantly lower proportion of normally hydrated patients received CRRT (*P* =0.80), died in the ICU (*P* =0.89) and in hospital (*P* =0.55) than dehydrated and overhydrated patients.

**Table 4:** Outcome data

	All	Dehydration	Normal	Overhydration	*P* value
	(*n* =61)	(*n* =14)	(*n* =22)	(*n* =25)	
Renal replacement therapy	8 (13 %)	2 (14 %)	2 (9 %)	4 (16 %)	0.80
ICU length of stay, days	5.1 (3.5–10)	6.1 (3.9–7.3)	4.7 (2.8–12)	7.0 (3.5–9.6)	0.87
Hospital length of stay, days	16 (7.9–30)	13 (7.6–21)	15 (7.3–32)	22 (11–36)	0.22
ICU mortality	7 (11 %)	2 (14 %)	2 (9 %)	3 (12 %)	0.89
Hospital mortality	12 (20 %)	4 (29 %)	3 (14 %)	5 (20 %)	0.55

*ICU* intensive care unit

Data are medians (interquartile range) or *n* (%)

## Discussion

### Key findings

We conducted a pilot, observational, clinician-blinded study of BIVA-measured hydration status in critically ill patients. We found that, on initial BIVA assessments, 40 % of patients were overhydrated and almost one-fourth were dehydrated. Patients with abnormal BIVA values had specific features congruent with BIVA measured status and appeared more severely ill than patients with normal BIVA status. Importantly, their subsequent mean cumulative fluid balance, as achieved by clinicians blinded to BIVA results, was positive for dehydrated patients, negative for overhydrated patients and neutral for normally hydrated patients. Moreover directional changes in BIVA were consistent with directional changes in fluid balance. Finally, BIVA correlated with cumulative fluid balance over the time in the ICU.

### Relationship to previous studies

To our knowledge, hydration status has been measured in ICU patients using BIVA technology in only three other studies [20–22]. Basso et al. reported a 70 % incidence of overhydration among 64 critically ill patients [21]. However, surprisingly, the hydration status of these patients remained largely unchanged during the observation period. Unfortunately, no separate information about dehydrated and normally hydrated patients was provided, and no data were presented on cumulative fluid balance in the different groups, to provide information on the validity of BIVA measurements.

In a previous study of 121 patients published in 2000 using older technology [20], single CVP and BIVA measurements were determined simultaneously. CVP values were significantly and inversely correlated with individual impedance vector components and with both vector components together. However, in this study, no additional BIVA measurements were obtained, and no information on changes in overall hydration status or fluid balance was provided.

In a recent study, fluid status was assessed using BIVA before the commencement of CRRT in 58 critically ill patients and 3 days afterward [22]. Measurements of serum N-terminal pro-B-type natriuretic peptide (NT-pro-BNP) were taken at the same time points. Patients were categorised as having either no overhydration or overhydration, but the specific BIVA cutoff values were not given and no separate information about dehydrated patients was provided. Similarly to our study, 50 % of patients were overhydrated before the start of CRRT according to the BIVA measurement and also had an abnormal NT-pro-BNP measurement. Cumulative fluid balance over 3 days was measured and was positive in all groups, but serial BIVA measurements over the same time frame were not obtained. Finally, in all the above studies, treating clinicians were not formally kept blinded to BIVA findings, creating an important risk of bias.

A relationship between fat-free TBW gain or loss and corresponding changes in BIVA hydration has been described in non-ICU patients. A 6.8-L TBW gain during pregnancy, quantified by deuterium dilution, was associated with a significant increase in BIVA hydration [10]. The sensitivity of BIVA to detect smaller hydration changes is uncertain, however. In the present study, we demonstrated an increase in BIVA hydration in patients with calculated fluid accumulations >1 L. Moreover, we observed a statistically significant decrease in BIVA hydration after a calculated median fluid loss of 2.4 L. This supports the results of a study by Piccoli and coworkers [17], who observed decreased hydration (increased vector length) in parallel with fluid removal of 2.4 L during haemodialysis.

### Implications of study findings

We have demonstrated the feasibility of performing repeated BIVA measurements in critically ill patients. Additionally, we have demonstrated that an abnormal BIVA value separates logical patient groups according to expected hydration status, that directional changes in BIVA hydration are consistent with directional changes in fluid balance, that changes in BIVA hydration are coherent with changes in fluid balance, and, finally that a change in BIVA over the duration of the ICU stay correlates with cumulative fluid balance. These observations support the face validity, concurrent validity, construct validity and content validity of BIVA as a measure of hydration in critically ill patients.

Our results suggest that BIVA may add useful information to guide fluid management in critically ill patients. However, although the directional changes in BIVA hydration corresponded with directional changes in cumulative fluid balance, a fluid loss >2 L was required for a corresponding decrease in BIVA hydration to reach statistical significance. This may imply that BIVA is insensitive to smaller fluid balance changes or, alternatively, reflects the imperfection of fluid balance calculations. Whether BIVA-guided fluid management improves patient-centred outcomes needs to be explored in future interventional studies.

Finally, we found a significant positive correlation between changes in lactate and cumulative fluid balance over the entire study period. Rather than being related to fluid status per se, this association is suggested to be related to illness severity because both increasing lactate and progressive fluid overload are typically observed in sicker patients. In fact, as demonstrated in Table 3, no significant changes in lactate during early fluid gains or losses >1–2 L were found. Moreover, we found no correlation between changes in lactate and changes in BIVA hydration.

### Strengths and limitations

Our study has several strengths. By obtaining twice-daily measurements over several consecutive days, we were able to exactly match changes in fluid balance with corresponding changes in BIVA hydration, CVP and lactate and thereby study their relationships. Since treating clinicians were blinded to the results, it was possible to do an unbiased comparison of patients’ characteristics and outcomes at different BIVA hydration levels. The observation that patients with dehydration, normal hydration and overhydration on initial BIVA assessment had different characteristics that could be expected owing to such differences in hydration status further supported our findings. Moreover, the fact that patients with dehydration, normal hydration and overhydration on initial BIVA assessment had a subsequent concordant change (positive, neutral or negative) in cumulative fluid balance supports the validity of BIVA. Finally, the observation that directional changes in BIVA hydration corresponded with directional changes in fluid balance lends robustness to our study.

This study has limitations. It is a single-centre study, with all the limitations inherent in such a design. However, we studied a heterogeneous population of ICU patients with >300 measurements, suggesting a degree of external validity and robustness. It is not an interventional study; therefore, we can make no inferences about the utility of BIVA in the management of fluid balance. However, observational studies such as this one are necessary to establish the feasibility, safety and validity of the technique before its application in interventional studies. We did not use the deuterium dilution method to confirm the accuracy of the fat-free TBW assessments provided by the BIVA technology. However, the lack of steady state in critically ill patients and the time taken for equilibration preclude the simultaneous use of gold standard isotopic tracers as a formal validating technique. In addition, the significant number of missing CVP values upon admission in dehydrated and normally hydrated patients needs to be acknowledged. This may have biased the relationships of CVP with BIVA hydration and cumulative fluid balance, respectively.

Whilst the BIVA measurements of hydration status mirror the changes seen in cumulative fluid balance, we acknowledge that the ICU environment itself may impact the accuracy of BIVA measurements. In some circumstances, patients could not be positioned completely supine (e.g., those with head injury and intracranial pressure monitoring), and on occasion the positioning of the electrodes had to be modified slightly owing to the presence of other devices (e.g., intravenous cannulae and soft restraints). The extensive electrical equipment in the ICU, including the various monitoring devices and mechanical ventilators, could potentially impact measured bioimpedance, as could any water in the patient’s bed, though to what degree is unknown. The fact that in this technically hostile environment BIVA still generated reproducible and logical findings supports the applicability of this technology in the ICU.

## Conclusions

BIVA hydration may be an additional measure of fluid status in critically ill patients. Two important findings support this conclusion. Firstly, patients with dehydration, normal hydration and overhydration at initial BIVA assessment had different characteristics that could be expected from such differences in hydration status. Secondly, patients with dehydration, normal hydration and overhydration in their initial BIVA assessment had subsequent changes (positive, neutral or negative) in cumulative fluid balance that were consistent with clinical expectations. However, our findings challenge the sensitivity of repeated BIVA hydration measurements as a dynamic indicator of changes in body water content. A relatively pronounced negative fluid balance was required for a significant corresponding directional change in BIVA hydration to occur. Moreover, the correlation between the overall change in BIVA hydration and the corresponding directional change in fluid balance was weak and of borderline significance. These conflicting findings support the need for further investigations of the utility of BIVA in critically ill patients.

## Key messages

- BIVA-measured hydration is an additional tool for fluid status assessment in critically ill patients and is feasible to use in such patients.
- Directional changes in BIVA were consistent with directional changes in fluid balance.
- BIVA measurements of hydration status correlated with cumulative fluid balance during the time in the ICU.
- The findings support that BIVA hydration is a valid measure of fluid status and justifies further studies to assess the utility of BIVA in critically ill patients.

## Additional file

Additional file 1: Figure S1. Fluid management. **Figure S2.** BIVA graphs in CRRT and ECMO patients. Assessment of repeatability. (DOCX 564 kb)

## Abbreviations

APACHE III: Acute Physiology and Chronic Health Evaluation
BIVA: Bioelectrical impedance vector analysis
COPD: Chronic obstructive pulmonary disease
CRRT: Continuous renal replacement therapy
CVP: Central venous pressure
FiO_2_: Fraction of inspired oxygen
H: Height
ICU: Intensive care unit
IQR: Interquartile range
MAP: Mean arterial pressure
NT-pro-BNP: N-terminal pro-B-type natriuretic peptide
PaO_2_: Partial pressure of arterial oxygen
R: Resistance
RM-ANOVA: Repeated-measures analysis of variance
TBW: Total body water
Xc: Reactance
