# Supplementary material for: Bioelectrical impedance vector analysis in critically ill patients: a prospective, clinician-blinded investigation
Source: Crit Care. 2015 Aug 12;19(1):290. doi: 10.1186/s13054-015-1009-3 (PMC4531396; doi:10.1186/s13054-015-1009-3)
Supplement: Additional file 1: Figure S1. — Fluid management. Figure S2. BIVA graphs in CRRT and ECMO patients. Assessment of repeatability. (DOCX 564 kb) [file 13054_2015_1009_MOESM1_ESM.docx]

**Bioelectrical Impedance Vector Analysis and Hydration status in Critically Ill Patients**

**Supplementary Material**


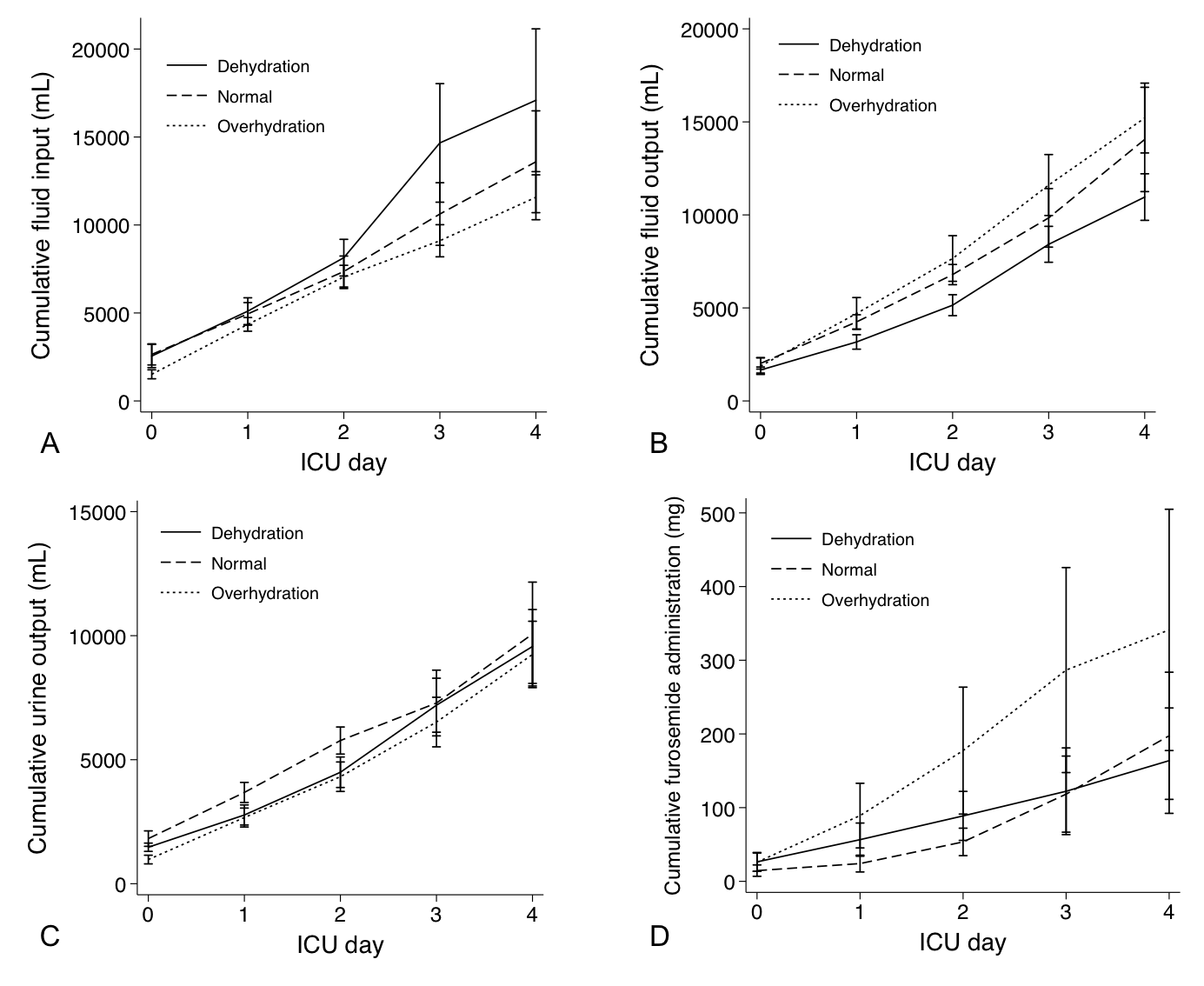


**Figure 1.** Cumulative fluid input (A), fluid output (B), urine output (C) and furosemide administration (D) during the first four days in ICU in patients with dehydration, normal hydration and overhydration on admission. Values are mean±SE.


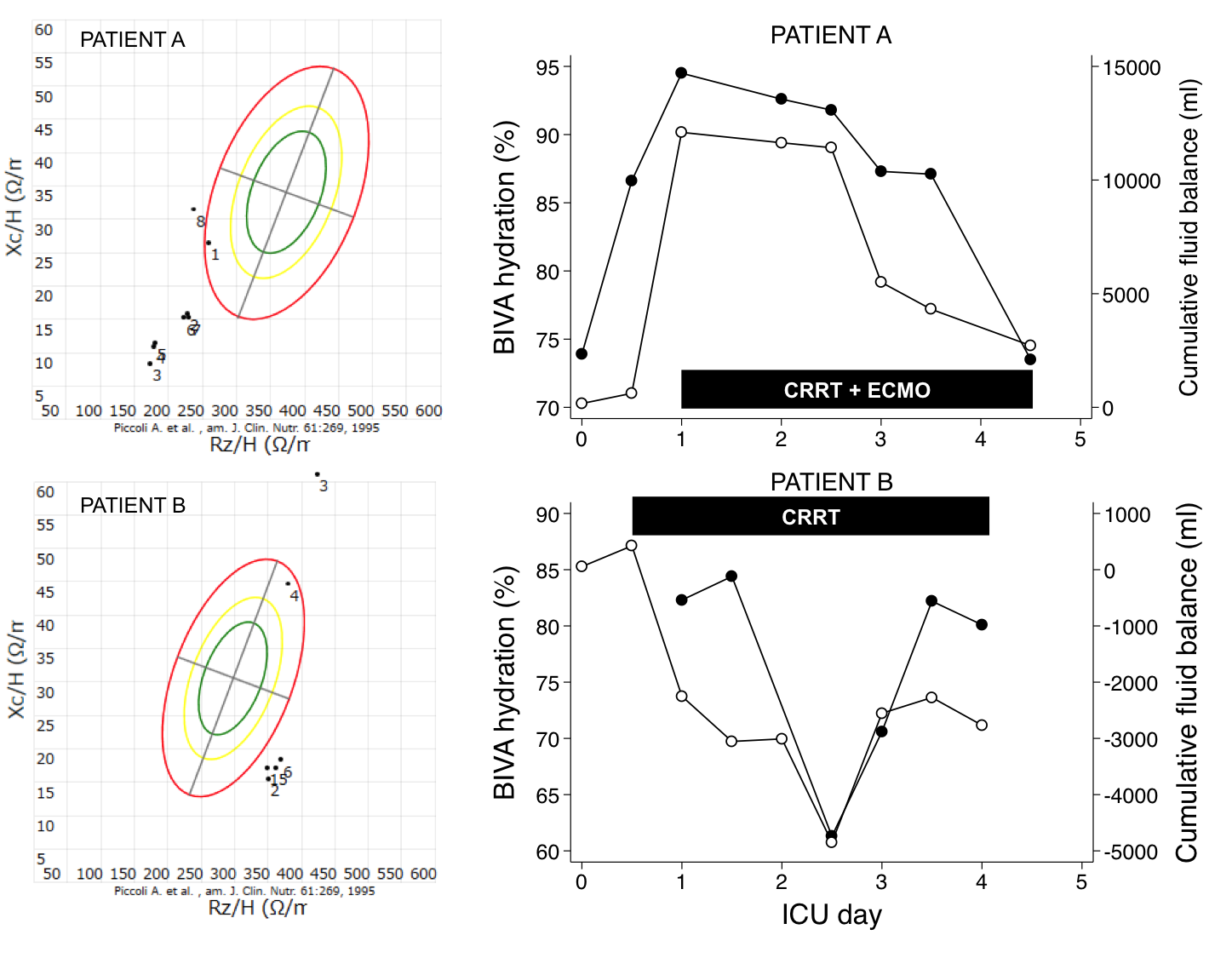


**Figure 2**. Bioelectrical impedance vector analysis (BIVA) graphs (left column) during ECMO and CRRT (patient A) and during CRRT only (patient B). The right column displays the corresponding BIVA hydration (closed circles) and cumulative fluid balance (open circles) during the four ICU days.

**Assessment of repeatability**

*Method*: We selected two consecutive BIVA-hydration readings per patient, obtained less than 24 hours apart, were the change in cumulative fluid balance between the two measurements was less than 100 ml to increase the likelihood of steady state between measurements. Coefficient of variation (%CV) for each patient was calculated by dividing the standard deviation of the two BIVA measurements by the duplicate mean, and multiplying by 100. The average of the individual CVs was reported as the intra-BIVA %CV.

*Results*: We identified 28 patients with such readings obtained 9.3 ± 4.1 hours apart. The mean change in fluid balance between the two readings in these patients was 42 ± 24 ml. The intra-BIVA %CV was 4.0, which suggests good precision.
